# In the heat of the moment: prevalence and clinical characteristics of headache associated with sexual activity (head HEATS study)

**DOI:** 10.1186/s10194-026-02516-7

**Published:** 2026-09-29

**Authors:** Simon Heintz, Florian Giese, Stefan Watzke, Dietrich Stoevesandt, Steffen Naegel, Torsten Kraya

**Affiliations:** 1 Department of Neurology, University Hospital Halle, Martin-Luther-University Halle- Wittenberg, Ernst-Grube-Straße 40, 06120 Halle (Saale), Germany; 2 Department of Neurology, Hospital St. Georg, Leipzig, Germany; 3 Department of Neurology, Alfried Krupp Krankenhaus Essen, Essen, Germany; 4 Department of Psychiatry, University Hospital Halle, Martin-Luther-University Halle- Wittenberg, Halle (Saale), Germany; 5 Department of Radiology, University Hospital Halle, Martin-Luther-University Halle- Wittenberg, Halle (Saale), Germany

**Keywords:** Headache associated with sexual activity, Primary headache disorder, ICHD-3, Epidemiology, Primary exercise headache

## Abstract

**Background:**

Headache associated with sexual activity (HAS) is an uncommon and understudied primary headache disorder. Previous epidemiological studies have estimated a lifetime prevalence of approximately 1.0–1.6%, although the true prevalence remains uncertain because symptoms related to sexual activity may be underreported.

**Objective:**

This study aimed to assess the prevalence and clinical characteristics of HAS and its co-occurrence with other primary headache disorders in a university-based cohort.

**Methods:**

A cross-sectional questionnaire study was conducted among students and employees at Martin- Luther-University Halle-Wittenberg, Germany. Of 1213 distributed questionnaires, 606 with complete age and sex information were included in the final analysis. Continuous variables were assessed for normality and compared using non-parametric methods when appropriate. Categorical variables were compared using two-sided Fisher’s exact tests. Associations with HAS were explored using parsimonious Firth penalized logistic regression models.

**Results:**

Twenty-three of 606 participants reported HAS, corresponding to a lifetime prevalence of 3.8% (95% CI, 2.6–5.6%). Participants with HAS were older than those without HAS (median [IQR], 25 [21–34.5] vs. 21 [19–26] years; *p* = 0.005). Although 82.6% of participants with HAS were female, the proportion did not differ significantly between groups (82.6% vs. 68.6%; *p* = 0.175). Among participants with available timing data, headache occurred during sexual arousal or the buildup to orgasm in 57.9% and at orgasm in 42.1%. Clinical characteristics were heterogeneous with respect to pain quality and localization, and symptoms compatible with migraine aura were reported by 34.7%. Migraine and tension-type headache were not significantly associated with HAS. In age- and sex-adjusted Firth penalized logistic regression, primary exercise headache was associated with higher odds of HAS (aOR 4.06, 95% CI 1.41–11.72; *p* = 0.010).

**Conclusions:**

In this university-based questionnaire study, 3.8% of participants reported symptoms compatible with HAS. The clinical presentation was heterogeneous, and primary exercise headache was associated with higher odds of HAS after adjustment for age and sex. Given the small number of HAS cases, questionnaire-based case ascertainment, and potential selection and response bias, these findings should be considered exploratory and should not be interpreted as population-representative prevalence estimates. Larger studies using clinician-confirmed diagnoses are needed to validate these findings.

**Supplementary Information:**

The online version contains supplementary material available at https://doi.org/10.1186/s10194-026-02516-7.

## Introduction

Headache associated with sexual activity (HAS) is a primary headache disorder classified in Chapter 4 of the International Classification of Headache Disorders under “Other Primary Headache Disorders” [1]. HAS is a unique condition characterized by the onset of headaches during sexual activity, either gradually increasing as sexual arousal progresses or abruptly intensifying at the moment of orgasm [2]. Although generally considered benign, these headaches can cause considerable distress and anxiety among affected individuals [3]. The ICHD-1 classification distinguishes between “dull”, “explosive at orgasm” and “postural and postcoital types” on the basis of headache location and pain characteristics and derives pathophysiological assumptions of those types. The introduction of a preorgasmic and/or orgasmic headache in the ICHD-2 classification has been removed in the latest version because the available body of clinical data could not support this distinction. The diagnostic criteria for HAS in the ICHD-3 classification include at least two episodes of pain in the head and/or neck, which fulfill the following criteria: (1) the headache is evolving and occurs only during sexual activity; (2) the pain in the head increases in intensity with increasing sexual arousal and/or has abrupt explosive intensity just before or with or during which the headache can occur; (3) the pain lasts from one minute to 24 hours with severe intensity and/or up to 72 hours with mild intensity; and (4) the pain cannot be better accounted for by another ICHD-3 diagnosis.

The available literature suggests that HAS has been underreported, with prior studies indicating a lifetime prevalence between 1.0% and 1.6% in the general population [3–5]. However, these figures are likely underestimated, as individuals may be reluctant to report headaches associated with intimate activities [6]. The stigma surrounding sexual health often prevents patients from seeking medical advice from healthcare providers, which may contribute to the low documented prevalence of HAS [4]. The lack of robust epidemiological data highlights the need for further investigations to accurately determine the prevalence and characteristics of HAS [7]. In contrast, the epidemiological data for other primary headache disorders, such as trigeminal autonomic cephalalgias (TAC), migraine, and tension-type headache (TTH), are already well-documented and provide a comprehensive understanding of their respective disease patterns [8].

A diagnosis does not require prior sexual intercourse, as cases of masturbation have also been reported in the literature [9]. Clinically, HAS presents with varying pain qualities, including dull, pressing, or explosive, and can be localized in different regions of the head, such as the occipital or temporal areas [2]. A recent narrative review further emphasized the heterogeneity of its clinical presentation and summarized the limited evidence regarding proposed pathophysiological mechanisms, diagnostic evaluation, and therapeutic approaches [10]. Importantly, particularly at first presentation, secondary causes of headache associated with sexual activity need to be considered and may require appropriate diagnostic evaluation [6, 10, 11].

The pain often lasts from a few minutes to a few hours, with some patients experiencing longer durations [9, 12].

The ICHD-3 classification provides diagnostic criteria for HAS, but owing to the rarity and limited number of properly scaled studies, there is still much to learn about the specific characteristics of these headaches [1]. Despite increasing clinical recognition of HAS, the available evidence remains limited. Previous research has largely consisted of case reports and small clinically characterized cohorts. For example, a case series of 13 patients with primary headache associated with sexual activity demonstrated variability in temporal presentation, pain localization, and co-occurring headache disorders [13]. While such clinical series provide valuable phenotypic information, their small and selected populations limit conclusions regarding prevalence and the distribution of clinical characteristics in broader populations [3].

Therefore, larger epidemiological studies using systematic assessment are needed to better characterize the prevalence and clinical spectrum of HAS. The present study aimed to assess the prevalence and clinical characteristics of HAS in a university-based cohort and to explore its co-occurrence with other primary headache disorders.

## Methods

### Study design

This study was designed as a cross-sectional survey to assess the prevalence and characteristics of headaches associated with sexual activity (HAS) in a large cohort predominantly comprising young adults. A structured questionnaire was developed to gather information on the occurrence, type, and associated factors of HAS, as well as its relationship with other primary headache disorders. The study was conducted in the Department of Neurology at Martin-Luther-University Halle-Wittenberg. This study is reported in accordance with the Strengthening the Reporting of Observational Studies in Epidemiology (STROBE) guidelines for cross-sectional studies. The completed STROBE checklist is provided as supplementary material (Additional file 3).

The 129-item questionnaire was developed by headache specialists certified by the German Migraine and Headache Society (DMKG) based on the ICHD-3 criteria and designed to ensure comprehensive coverage of relevant diagnostic features. Each item was specifically designed to operationalize the four diagnostic criteria for HAS as defined by the ICHD-3: (1) onset during sexual activity, (2) pain pattern relative to sexual arousal/orgasm, (3) pain duration and intensity, and (4) exclusion of other headache disorders considering the patient’s history. The questionnaire was developed by headache specialists, and its design and implementation have previously been documented within the framework of a doctoral thesis [14]. The original German questionnaire and an English translation are provided as Additional files 1 and 2, respectively.

### Ethical considerations

The study protocol was approved by the Ethics Committee of Martin-Luther-University Halle-Wittenberg. Ethical approval was obtained on January 20, 2015, with the requirement that potentially affected participants be provided with an opportunity for follow-up medical consultations if necessary. The participants were provided with email contact for further assistance. The study strictly adhered to ethical guidelines concerning participant confidentiality, informed consent, and the voluntary nature of participation.

### Cohort

The study cohort consisted of students and employees of Martin-Luther-University Halle Wittenberg. The participants were recruited through educational events and university networks. The participants were required to provide basic demographic information (age, sex) and details regarding their experience with primary headaches, including those associated with sexual activity. A total of 1213 questionnaires were distributed. The exclusion criteria were incomplete questionnaires or failure to provide age and sex information.

### Questionnaire

A standardized questionnaire was developed to capture data related to HAS and other primary headache disorders. The questionnaire comprised 129 items divided into three sections:

*Demographic Data and Headache History* (39 items): This section collected data on age, sex, the prevalence of known primary headaches (e.g., migraine, tension-type headache), the frequency of headaches, and the family history of headaches.

*Characteristics of HAS* (42 items): Participants were asked about their experience with HAS, including age at onset, frequency, pain intensity (on a numerical rating scale, NRS), pain duration, pain quality (e.g., dull, pressing, stabbing), and the presence of any accompanying symptoms such as nausea, visual disturbances, or trigeminal autonomic symptoms.

*Co-occurrence with other headache disorders* (48 items): This section focused on identifying any co-occurring primary headaches, such as migraine, tension-type headache (TTH), or primary exercise headache. It also explored family history and other possible triggering factors.

The questionnaire also assessed the use of selected headache-related medications, including beta-blockers, flunarizine, topiramate, amitriptyline, valproate, triptans, and commonly used analgesics. PDE5 inhibitor use was not specifically assessed.

The questionnaire was designed to be self-administered, with clear instructions provided to minimize errors. It was distributed primarily during university lectures to maximize participation and control over the distribution process. Electronic versions of the questionnaires were created using the EvaSys system (EvaSys V7.1, Electric Paper Evaluationssysteme GmbH, Lüneburg, Germany), which also facilitated the automated scanning and electronic analysis of responses.

### Data collection and analysis

Data were collected electronically through the EvaSys platform, which allowed for automatic reading and recording of responses. Responses were processed, and incomplete or ambiguous answers were flagged for manual review. To ensure data integrity, only questionnaires with complete demographic information on age and sex were included in the final analytical cohort. Questionnaires with missing age and/or sex information were excluded in accordance with the study protocol.

The collected data were exported to IBM SPSS Statistics Version 22 for statistical analysis. Continuous variables were assessed for normality using the Shapiro–Wilk test and inspection of their distributions. Normally distributed continuous variables are presented as mean ± standard deviation (SD), whereas non-normally distributed variables are reported as median and interquartile range (IQR). As age was not normally distributed, differences in age between participants with and without HAS were assessed using the Mann–Whitney U test.

Categorical variables are reported as absolute frequencies and percentages and were compared using the chi-square test or Fisher’s exact test, as appropriate.

To assess the robustness of the age-related findings, two post hoc sensitivity analyses were performed. First, the age comparison was repeated after excluding participants younger than 18 years. Second, the age comparison was repeated after excluding participants with age values more than 2.5 standard deviations above or below the mean age of the final analytical cohort.

To limit model overfitting, regression analyses were performed using a parsimonious modelling strategy. A minimally adjusted Firth penalized logistic regression model including age and sex was fitted first. In separate subsequent models, migraine, tension-type headache, and primary exercise headache were each added individually to the age- and sex-adjusted model. Analyses involving these additional variables were based on participants with available data for the respective variable. Adjusted odds ratios (aOR) with 95% confidence intervals (CI) are reported.

The lifetime prevalence of HAS is reported with a 95% confidence interval (CI) calculated using the Wilson score method. All statistical tests were two-sided, and a *p* value < 0.05 was considered statistically significant. Given the exploratory nature of the study, no formal adjustment for multiple testing was applied.

## Results

### Sample characteristics

**Fig. 1:**
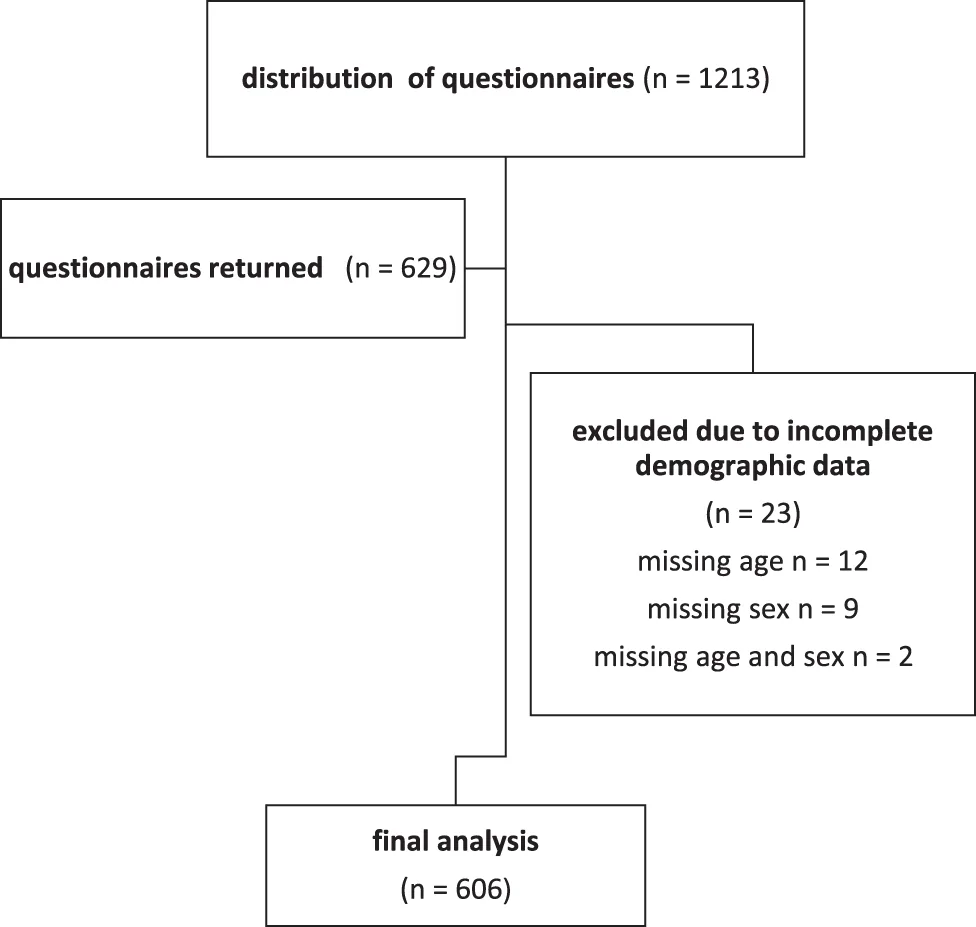
Flow chart of data collection and analysis

Of the 1213 questionnaires distributed, 629 were returned, corresponding to a response rate of 51.9%. Of these, 23 questionnaires were excluded because of missing demographic information required for the primary analyses (missing age, *n* = 12; missing sex, *n* = 9; missing age and sex, *n* = 2), resulting in a final analytical cohort of 606 participants (Fig. 1). The median age of the final analytical cohort was 21 years (IQR 19–26; range, 17–63 years), and 419 participants (69.1%) were female.

The results of both post hoc sensitivity analyses were consistent with the primary analysis. When restricting the analysis to adult participants aged 18 years or older, 543 participants remained (HAS, *n* = 22; no HAS, *n* = 521). Participants with HAS had a higher median age than those without HAS (median [IQR], 25.5 [21–34.75] vs. 22 [19–26] years; *p* = 0.008). In a separate sensitivity analysis excluding participants with age values more than 2.5 standard deviations from the mean age of the final analytical cohort, 586 participants remained (HAS, *n* = 21; no HAS, *n* = 565). Participants with HAS again had a higher median age than those without HAS (median [IQR], 23 [21–34] vs. 21 [19–25] years; *p* = 0.012).

### Prevalence of headaches associated with sexual activity (HAS)

Among 606 participants included in the final analysis, 23 reported HAS, corresponding to a lifetime prevalence of 3.8% (95% CI, 2.6–5.6%). Participants with HAS were older than those without HAS (median [IQR], 25 [21–34.5] vs. 21 [19–26] years; *p* = 0.005). Most individuals with HAS were female (82.6%, *n* = 19), although the proportion of female participants did not differ significantly between participants with and without HAS (82.6% vs. 68.6%; *p* = 0.175). The baseline demographic and headache characteristics of the participants with and without HAS are summarized in Table 1.

**Table 1:** Baseline characteristics of participants with and without headache associated with sexual activity (HAS). Data are presented as median [IQR] or n (%), as appropriate. Age was compared using the mann–Whitney U test; categorical variables were compared using two-sided Fisher’s exact tests. IQR, interquartile range

Characteristic	HAS (n = 23)	No HAS (n = 583)	p value
Age, years, median [IQR]	25 [21–34.5]	21 [19–26]	0.005
Female sex, n (%)	19 (82.6)	400 (68.6)	0.175
Migraine, n (%)	5 (21.7)	90 (15.4)	0.385
Tension-type headache, n (%)	8 (34.8)	139 (23.8)	0.223
Primary exercise headache, n (%)	5 (21.7)	37 (6.3)	0.017

### Characteristics of HAS

**Fig. 2:**
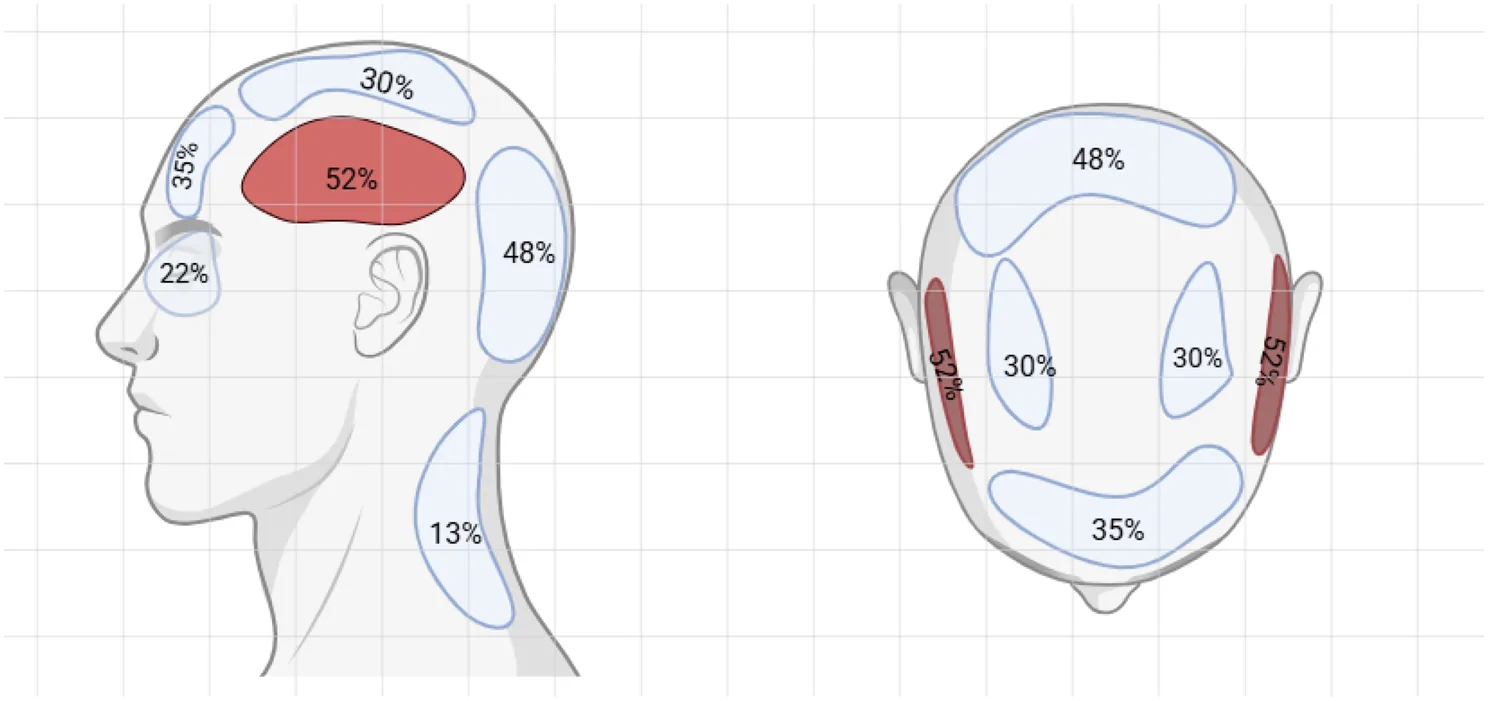
Anatomical distribution of pain locations in participants with headache associated with sexual activity (HAS). Created with BioRender®

Clinical characteristics of HAS are summarized in Table 2. Among participants with available timing data, 57.9% (11/19) reported headache during sexual arousal or the buildup to orgasm, whereas 42.1% (8/19) reported headache at orgasm. Most participants with available duration data reported headache lasting up to 1 hour (77.3%, 17/22). Pain characteristics were heterogeneous. Temporal and occipital regions were the most frequently reported pain locations (Fig. 2), whereas dull/pressing pain was the most common pain quality. Symptoms compatible with migraine aura were reported by 34.7% (8/23) of participants.

**Table 2:** Clinical characteristics of participants with headache associated with sexual activity (HAS). Data are presented as n/N (%) where appropriate. NRS, numerical rating scale

Clinical characteristic	HAS participants
*Timing of headache*	
During sexual arousal/pre-orgasmic	11/19 (57.9%)
At orgasm	8/19 (42.1%)
*Onset*	
Gradual onset among pre-orgasmic headaches	9/11 (81.8%)
Explosive onset among pre-orgasmic headaches	2/11 (18.2%)
Sudden/explosive onset among orgasm-associated headaches	8/8 (100%)
*Duration*	
1 min	7/22 (31.8%)
30 min − 1 h	10/22 (45.5%)
1–12 h	5/22 (22.7%)
12–24 h	0/22 (0%)
24–72 h	0/22 (0%)
*Maximum pain intensity*	
Mild (NRS 1–5/10)	7/21 (33.3%)
Severe (NRS > 5/10)	14/21 (66.7%)
*Pain quality*	
Dull/pressing	8/23 (34.8%)
Stabbing	5/23 (21.7%)
Pulsating	4/23 (17.4%)
*Associated symptoms*†	
Symptoms compatible with migraine aura	8/23 (34.7%)
Trigeminal autonomic symptoms: Rhinorrhea	2/23 (8.7%)
Tearing	1/23 (4.3%)

†Multiple responses were possible; therefore, percentages do not necessarily sum to 100%

### Co-occurrence with other primary headache disorders

The present study explored the co-occurrence of HAS with other primary headache disorders, including migraine, tension-type headache (TTH), and primary exercise headache.

*Migraine*: Migraine was reported by 21.7% (*n* = 5) of participants with HAS and by 15.4% (*n* = 90) of participants without HAS. No statistically significant association between HAS and migraine was observed (*p* = 0.385; OR 1.52).

*Tension-Type Headache* (TTH): TTH was reported by 34.8% (*n* = 8) of participants with HAS and by 23.8% (*n* = 139) of participants without HAS. No statistically significant association between HAS and TTH was observed (*p* = 0.223; OR 1.70).

*Primary Exercise Headache*: Primary exercise headache was reported by 21.7% (*n* = 5) of participants with HAS compared with 6.3% (*n* = 37) of participants without HAS (*p* = 0.017; OR 4.10).

#### Pain intensity and co-occurring primary headache disorders

Pain intensity did not differ significantly according to the presence of co-occurring migraine, TTH, or primary exercise headache in exploratory non-parametric comparisons (all *p* > 0.05). Given the small numbers within these subgroups, these analyses should be interpreted cautiously.

In the minimally adjusted model including age and sex, increasing age was associated with higher odds of HAS (aOR 1.06 per year, 95% CI 1.03–1.10; *p* < 0.001), whereas female sex was not significantly associated with HAS (aOR 2.10, 95% CI 0.74–5.96; *p* = 0.164). The association with age remained consistent when migraine, TTH, or primary exercise headache were added individually to the age- and sex-adjusted model. Neither migraine nor TTH was significantly associated with HAS. In contrast, primary exercise headache was associated with higher odds of HAS after adjustment for age and sex (aOR 4.06, 95% CI 1.41–11.72; *p* = 0.010; Table 3).

**Table 3:** Firth penalized logistic regression analyses of factors associated with headache associated with sexual activity (HAS). Model 1 included age and sex. Models 2–4 additionally included migraine, tension-type headache (TTH), and primary exercise headache, respectively. The overall analytical cohort comprised 606 participants. Models 2–4 were based on complete cases for the additional headache variable included in the respective model, resulting in model-specific sample sizes. Values are presented as adjusted odds ratios (aOR) with 95% confidence intervals (CI) and *p* values. An em dash (—) indicates that the variable was not included in the respective model

Variable	Model 1	Model 2	Model 3	Model 4
Age, per year	1.06 (1.03–1.10), *p* < 0.001	1.05 (1.01–1.09), *p* = 0.008	1.06 (1.02–1.10), *p* = 0.002	1.06 (1.02–1.10), *p* = 0.002
Female sex	2.10 (0.74–5.96), *p* = 0.164	2.00 (0.70–5.70), *p* = 0.192	2.45 (0.77–7.84), *p* = 0.130	2.48 (0.78–7.92), *p* = 0.125
Migraine	—	1.42 (0.53–3.83), *p* = 0.486	—	—
TTH	—	—	1.58 (0.65–3.85), *p* = 0.317	—
Primary exercise headache	—	—	—	4.06 (1.41–11.72), *p* = 0.010
Participants/HAS cases, n	606/23	577/22	564/21	550/21

No statistically significant associations were observed for female sex, migraine, or TTH (Table 3).

## Discussion

This cross-sectional questionnaire study provides additional data on the prevalence and clinical characteristics of headache associated with sexual activity (HAS). The observed lifetime prevalence was 3.8% (95% CI, 2.6–5.6%), which is higher than the 1.0–1.6% reported in previous epidemiological studies [2–5, 15]. However, this estimate should be interpreted cautiously given the university-based study population, voluntary participation, questionnaire-based case ascertainment, and the limited number of participants reporting HAS. Participants with HAS showed a heterogeneous clinical phenotype, and primary exercise headache was associated with higher odds of HAS after adjustment for age and sex. Given the exploratory design and the small number of HAS cases, the present findings should primarily be considered hypothesis-generating.

### Prevalence and demographic characteristics

The observed lifetime prevalence of 3.8% should not be interpreted as a population-based prevalence estimate. Participation was voluntary, and the sensitive nature of questions concerning sexual activity may have introduced both self-selection and response bias. Individuals with relevant headache experiences may have been more likely to participate or report symptoms, potentially resulting in overestimation. Conversely, reluctance to disclose symptoms occurring during sexual activity may have resulted in underreporting. The direction and magnitude of these potential biases cannot be determined from the present data.

Accordingly, the higher prevalence observed in our cohort compared with previous estimates should be interpreted cautiously and may reflect differences in study population, recruitment, and case ascertainment rather than a true epidemiological difference. Although the anonymous questionnaire format may have facilitated disclosure of symptoms related to sexual activity [6, 16], anonymity alone cannot eliminate selection or response bias. Population-based studies using standardized diagnostic procedures are needed to provide more robust prevalence estimates.

Although most participants with HAS were female, no statistically significant association between female sex and HAS was observed. Given the predominance of women in the overall study population and the small number of HAS cases, our findings neither confirm nor refute the male predominance described in previous reports [17]. Larger and more representative cohorts are required to characterize potential sex differences in HAS prevalence.

### Clinical phenotype and diagnostic considerations

The clinical presentation of HAS was heterogeneous. Headaches were reported both during increasing sexual arousal and abruptly at orgasm, consistent with variation in the temporal presentation of HAS. Although the current ICHD-3 classification incorporates these presentations within a single diagnostic entity [1], the clinical relevance of different temporal patterns remains uncertain. Whether such patterns are associated with differences in clinical course or treatment response should be evaluated in prospective, clinician-based studies.

Participants also reported variation in pain quality and anatomical distribution, with temporal and occipital locations most frequently reported and dull/pressing pain being the most common pain quality. These observations provide descriptive information on the range of symptoms reported by participants with HAS. However, given the small number of cases and reliance on questionnaire-based symptom characterization, they should not be interpreted as evidence for modification of the current diagnostic criteria. Prospective studies with clinician-confirmed diagnoses are required to determine the reproducibility and clinical relevance of these phenotypic characteristics.

Some participants also reported symptoms compatible with migraine aura and trigeminoautonomic symptoms. Whether these manifestations represent features of HAS, overlap with other primary headache disorders, or limitations in questionnaire-based symptom attribution cannot be determined from the present study. Their potential clinical significance therefore requires confirmation using standardized neurological assessment.

### Co-occurrence with other primary headache disorders

We did not observe statistically significant associations between HAS and either migraine or tension-type headache (TTH). Previous studies have reported co-occurrence of HAS with other primary headache disorders, particularly migraine [10, 13]. For example, migraine was reported in 5 of 13 patients in a clinical case series [13].

However, the absence of statistically significant associations should not be interpreted as evidence of absence, particularly given the small number of HAS cases and the resulting limited statistical precision.

An additional limitation is that migraine and TTH were based on participants’ questionnaire responses rather than diagnoses established through a clinical interview. Consequently, misclassification of these headache disorders cannot be excluded. This limitation should be considered when interpreting both their observed frequencies and their associations with HAS.

In contrast, primary exercise headache was associated with higher odds of HAS after adjustment for age and sex (aOR 4.06, 95% CI 1.41–11.72). This finding remained evident in the parsimonious regression approach used to reduce the risk of model overfitting. Nevertheless, the wide confidence interval reflects considerable uncertainty, and the association is based on a small number of participants with both conditions. It should therefore be regarded as exploratory rather than confirmatory. Both headache disorders occur in relation to activities involving physiological exertion, making further investigation of a possible relationship of clinical interest. Larger prospective studies are needed to determine whether the observed association is reproducible and whether shared mechanisms are involved.

### Strengths and limitations

Several limitations should be considered when interpreting the findings. First, HAS was identified through a structured self-administered questionnaire rather than a clinician-confirmed diagnosis. Although the questionnaire was designed to capture symptoms compatible with the ICHD-3 criteria, questionnaire-based assessment cannot replace a comprehensive neurological evaluation and does not permit reliable exclusion of secondary causes of headache associated with sexual activity. The present study should therefore be regarded as describing self-reported symptoms compatible with HAS rather than providing definitive diagnostic confirmation.

Second, voluntary participation and the sensitive nature of the study topic introduce the possibility of self-selection, response, and reporting bias. These biases may have affected both the estimated prevalence and the reported clinical characteristics, and their direction and magnitude cannot be determined from the available data.

Third, migraine and TTH were assessed by questionnaire rather than confirmed by specialist interview, introducing the possibility of diagnostic misclassification. Accordingly, analyses concerning the co-occurrence of these headache disorders should be interpreted cautiously.

Fourth, although selected headache-related medications were assessed, none of the participants with HAS and available medication data reported use of the assessed preventive migraine medications. However, PDE5 inhibitor use was not specifically recorded, and residual confounding related to unmeasured medication exposure cannot be excluded.

Fifth, the university-based cohort consisted predominantly of young adults and included a higher proportion of female participants, limiting generalizability to the broader population. Although sensitivity analyses addressing age restrictions and age outliers yielded consistent age-related findings, these analyses do not overcome the limited representativeness of the study population.

Finally, only 23 participants reported HAS. This limited the statistical precision of subgroup comparisons and multivariable analyses. We therefore used parsimonious Firth penalized regression models and regard the resulting associations as exploratory. The cross-sectional design additionally precludes conclusions regarding causality or the temporal relationship between HAS and co-occurring headache disorders.

Despite these limitations, the anonymous study design enabled systematic investigation of a sensitive and understudied headache disorder in a relatively large questionnaire-based cohort. The study also provides descriptive data that may inform the design of future population-based and clinically validated investigations.

## Conclusions

This study provides additional epidemiological and phenotypic data on self-reported symptoms compatible with HAS in a university-based cohort. The observed lifetime prevalence was 3.8%, although this estimate should be interpreted in the context of potential selection and response bias and should not be considered population-representative. HAS showed heterogeneous temporal and clinical presentations, and primary exercise headache was associated with higher odds of HAS after adjustment for age and sex. Given the questionnaire-based case ascertainment and the small number of HAS cases, these findings are exploratory and hypothesis-generating. Future studies in larger and more representative populations, incorporating clinician-confirmed ICHD-3 diagnoses, exclusion of secondary causes, and systematic assessment of medication exposure, are needed to validate these observations and further characterize the clinical spectrum of HAS.

## Electronic supplementary material

Below is the link to the electronic supplementary material.

Supplementary Material 1: Original version of the questionnaire

Supplementary Material 2: Translation of the questionnaire

Supplementary Material 3: STROBE checklist

## Data Availability

The raw data generated and analyzed during the current study are not publicly available. The study protocol was approved by the Ethics Committee of the University Hospital Halle (reference 2014-147). The raw data are available from the corresponding author upon reasonable request.

## Abbreviations

aOR: Adjusted odds ratio
CI: Confidence interval
DMKG: German Migraine and Headache Society
HAS: Headache associated with sexual activity
ICHD: International Classification of Headache Disorders (ICHD-1, ICHD-2, and ICHD-3 denote the respective editions)
IQR: Interquartile range
NRS: Numerical rating scale
OR: Odds ratio
PDE5: Phosphodiesterase type 5
SD: Standard deviation
TTH: Tension-type headache
