# Supplementary material for: In the heat of the moment: prevalence and clinical characteristics of headache associated with sexual activity (head HEATS study)
Source: J Headache Pain. 2026 Sep 29;27(1):228. doi: 10.1186/s10194-026-02516-7 (PMC13625492; doi:10.1186/s10194-026-02516-7)
Supplement: Supplementary file 1 [file 10194_2026_2516_MOESM1_ESM.pdf]

Studie: Bestimmung von Häufigkeit, Charakteristika und möglicher pathophysiologischer Zusammenhänge seltener primärer Kopfschmerzformen

Klinik und Poliklinik für Neurologie Direktor: Prof. Dr. Stephan Zierz

Markieren Sie so: ☐ ☒ ☐ ☐ ☐ Bitte verwenden Sie einen Kugelschreiber oder nicht zu starken Filzstift. Dieser Fragebogen wird maschinell erfasst.
Korrektur: ☐ ■ ☐ ☒ ☐ Bitte beachten Sie im Interesse einer optimalen Datenerfassung die links gegebenen Hinweise beim Ausfüllen.

1. Allgemeines

Sehr geehrte Damen und Herren,

mit dem folgenden anonymen Fragebogen möchten wir Sie im Rahmen eines Promotionsvorhabens zur Häufigkeit von primären Kopfschmerzen befragen. Dabei sollen der Kopfschmerz bei sexueller Aktivität und der Eiskremkopfschmerz im Vordergrund stehen. Wir erhoffen uns von Ihren Angaben die Häufigkeit und Ursachen dieser Kopfschmerzformen besser verstehen zu können. Die Teilnahme an der Befragung ist freiwillig. Es handelt sich um einen anonymen Fragebogen dessen Daten in der Universitätsklinik für Neurologie gesammelt und ausgewertet werden. Bei Veröffentlichung der Studienergebnisse können keinerlei Rückschlüsse auf Ihre Person gezogen werden. Mit dem Ausfüllen des Fragebogens und der Abgabe oder Zusendung erklären Sie sich damit einverstanden, dass Ihre Angaben gesammelt und wissenschaftlich ausgewertet werden.
Bitte füllen Sie den Fragebogen nur einmal aus um die Ergebnisse der Studie nicht zu verfälschen.
Sollten Sie Rückfragen zu dieser Untersuchung haben können Sie uns jederzeit kontaktieren. Wir danken für Ihre Kooperation.

(Die zuständige Ethikkommission der Medizinischen Fakultät der Martin-Luther-Universität Halle-Wittenberg hat keine Einwände gegen die Durchführung dieser Studie erhoben.)

Mit freundlichen Grüßen,

Dr. med. Torsten Kraya Oberarzt Leiter der Kopfschmerzambulanz
Universitätsklinik und Poliklinik für Neurologie der Martin-Luther-Universität Halle-Wittenberg
Ernst-Grube-Str. 40 06097 Halle (Saale)
Tel.: (0345) 5 57 3340
Fax: (0345) 5 57 3335

1.1 **Bitte codieren Sie Ihr momentanes Alter wie in der Einleitung erklärt.**

10er ☐ ☐ ☐ ☐ ☐ ☐ ☐ ☐ ☐ ☐
1er ☐ ☐ ☐ ☐ ☐ ☐ ☐ ☐ ☐ ☐
x0 x1 x2 x3 x4 x5 x6 x7 x8 x9

1.2 **Wie ist ihr biologisches Geschlecht?** ☐ Weiblich ☐ Männlich

1.3 **Haben Sie regelmäßig gleichartige Kopfschmerzen?**
☐ Nie ☐ Sehr selten (1 mal in 3 Monaten) ☐ Selten (1-6 mal im Monat)
☐ Häufig (7-15 mal im Monat) ☐ Täglich

Leiden Sie <u>bekannterweise</u> unter einer der aufgeführten Kopfschmerzformen?

1.4	Migräne	☐ Ja	☐ Nein
1.5	Clusterkopfschmerz	☐ Ja	☐ Nein
1.6	Hustenkopfschmerz	☐ Ja	☐ Nein
1.7	Spannungskopfschmerz	☐ Ja	☐ Nein
1.8	Trigeminusneuralgie	☐ Ja	☐ Nein
1.9	Kopfschmerz bei körperlicher Belastung	☐ Ja	☐ Nein
1.10	SUNCT- Kopfschmerz	☐ Ja	☐ Nein
1.11	Thunderclap- Kopfschmerz (Donnerschlag)	☐ Ja	☐ Nein
1.12	Paroxysmale Hemikranie	☐ Ja	☐ Nein

1. Allgemeines [Fortsetzung]

1.13 **Seit wann leiden Sie an einem der o.g. Kopfschmerzen?**
☐ Ich leide unter keiner der o.g. Kopfschmerzformen ☐ Seit dem Erinnerungen bestehen (ca. 6. LJ) ☐ Seit dem Übertritt in das Pubertätsalter (ca. 12. LJ)
☐ Seit dem Verlassen der Pubertät (ca. 18. LJ)

Leidet eines Ihrer Familienmitglieder an einer primären Kopfschmerzform?

1.14	Mutter	☐ Ja	☐ Nein	☐ Weiß nicht
1.15	Vater	☐ Ja	☐ Nein	☐ Weiß nicht
1.16	Schwester	☐ Ja	☐ Nein	☐ Weiß nicht
1.17	Bruder	☐ Ja	☐ Nein	☐ Weiß nicht
1.18	Großmutter	☐ Ja	☐ Nein	☐ Weiß nicht
1.19	Großvater	☐ Ja	☐ Nein	☐ Weiß nicht

Leiden Sie <u>bekannterweise</u> unter einer der folgenden Erkrankungen?

1.20	Asthma	☐ Ja	☐ Nein
1.21	Koronare Herzkrankheit	☐ Ja	☐ Nein
1.22	Depressionen	☐ Ja	☐ Nein
1.23	Malignome (Krebserkrankungen)	☐ Ja	☐ Nein
1.24	Arterieller Hypertonus (Bluthochdruck)	☐ Ja	☐ Nein
1.25	Angsterkrankung	☐ Ja	☐ Nein

Nehmen Sie zur <u>Vorbeugung</u> einer Migräneattacke eines der folgenden Medikamente ein?

1.26	Betablocker (Metoprolol/ Propanolol)	☐ Ja	☐ Nein
1.27	Flunarizin (Flunavert/ Natil N)	☐ Ja	☐ Nein
1.28	Topiramat (Topimac/ Topimax/ Topiragamma)	☐ Ja	☐ Nein
1.29	Amitriptylin (Amineurin/ Saroten/ Syneudon)	☐ Ja	☐ Nein
1.30	Valporinsäure (Orfiril)	☐ Ja	☐ Nein

Nehmen Sie in der <u>Akutphase des Kopfschmerzes</u> eines/ mehrere der folgenden <u>Schmerzmedikamente</u> ein?

1.31	Aspirin®/ ASS	☐ Ja	☐ Nein
1.32	Ibuprofen (Dolormin®/ Ibudolor®/ Dolgit®)	☐ Ja	☐ Nein
1.33	Naproxen	☐ Ja	☐ Nein
1.34	Diclofenac (Diclac®/ Voltaren®)	☐ Ja	☐ Nein
1.35	Phenazon (Migräne-Kranit®)	☐ Ja	☐ Nein
1.36	Metamizol (Novalgin®/ Novaminsulfon)	☐ Ja	☐ Nein
1.37	Paracetamol	☐ Ja	☐ Nein
1.38	ASS+Paracetamol+Coffein (Thomapyrin®/ Dolopyrin®/ Mipyrin®/ Neuralgin®/ Ratiopyrin®)	☐ Ja	☐ Nein
1.39	Triptane (Sumatriptan/ Eletriptan/ o.Ä.)	☐ Ja	☐ Nein

2. Spezieller Teil: Kopfschmerz bei sexueller Aktivität

2.1 **Haben Sie Kopfschmerzen vor, während oder nachdem Sie Geschlechtsverkehr hatten?** ☐ Nie ☐ Immer ☐ Gelegentlich

Wenn Sie hier mit "Nie" antworten, blättern Sie bitte weiter zum Teil "Eiscremekopfschmerz" auf Seite 4

3. Eiscremekopfschmerz (Kopfschmerz nach Ingestition eiskalter Nahrungsmittel) [Fortsetzung]

3.9 Hängt das Auftreten des Kopfschmerzes mit der verzehrten **Menge** und/oder mit der **Geschwindigkeit** des Verzehrs zusammen? (bitte nur **EINE** Antwort ankreuzen)

☐ Menge und Geschwindigkeit haben **keinen** Einfluss auf den Kopfschmerz
☐ Der Kopfschmerz tritt bei **raschem** Verzehr **großer** Mengen auf
☐ Der Kopfschmerz tritt bei **raschem** Verzehr **kleiner** Mengen auf
☐ Der Kopfschmerz tritt bei **langsamem** Verzehr **großer** Mengen auf
☐ Der Kopfschmerz tritt bei **langsamem** Verzehr **kleiner** Mengen auf

3.10 Wie **intensiv** ist dieser Kopfschmerz auf einer Skala von 1-10? (1= leichtester, 10= Vernichtungsschmerz)
1 ☐ ☐ ☐ ☐ ☐ ☐ ☐ ☐ ☐ ☐ 10 ☐ Weiß nicht

3.11 Mit welcher der folgenden **Schmerzqualitäten** zeigt sich der Kopfschmerz nach Verzehr eiskalter Nahrungsmittel bei Ihnen am ehesten? (bitte nur **EINE** Antwort ankreuzen)
☐ Brennend ☐ Stechend ☐ Dumpf/ Drückend
☐ Ziehend ☐ Pulsierend ☐ Hämmernd
☐ Bohrend ☐ Weiß nicht ☐ Mit keiner der genannten

3.12 Wie lange dauert der Kopfschmerz nach dem Verzehr eiskalter Nahrungsmittel in den meisten Fällen an? (bitte nur **EINE** Antwort ankreuzen)
☐ **kürzer** als 10 Sekunden ☐ ca. 15-30 Sekunden ☐ **länger** als <u>eine</u> Minute
☐ **länger** als <u>10</u> Minuten ☐ Weiß nicht

3.13 Tritt der Kopfschmerz nach Verzehr eiskalter Nahrungsmittel stets ein- oder beidseitig auf? (bitte nur **EINE** Antwort ankreuzen)
☐ Kopfschmerz ist **stets einseitig** ☐ Kopfschmerz ist **stets beidseitig** ☐ Immer **unterschiedlich**

3.14 Tritt der Kopfschmerz nach Verzehr eiskalter Nahrungsmittel, bei wiederholtem Auftreten, immer auf der selben Seite und am gleichen Punkt des Schädels auf? (bitte nur **EINE** Antwort ankreuzen)

☐ Seite **und** genauer Punkt sind immer **gleich**
☐ Kopfschmerz **wechselt** die Seite, ist aber am **gleichen** Punkt wie auf der Gegenseite
☐ Kopfschmerz bleibt zwar auf der **selben** Seite, tritt jedoch immer an einem **anderen** Punkt auf
☐ Kopfschmerz tritt immer an **unterschiedlichen** Punkten **und** Seiten des Schädels auf

Wo, <u>außen auf den Schädel projiziert</u>, treten die Kopfschmerzen nach Verzehr eiskalter Nahrungsmittel auf? Die Kopfschmerzen treten auf im Bereich des/der...

3.15	Hinterkopfes	☐ Ja	☐ Nein
3.16	Nackens	☐ Ja	☐ Nein
3.17	Scheitels	☐ Ja	☐ Nein
3.18	Schläfen	☐ Ja	☐ Nein
3.19	Stirn	☐ Ja	☐ Nein
3.20	Oberer Nasenbereich/ Nasenwurzel	☐ Ja	☐ Nein
3.21	Augen	☐ Ja	☐ Nein
3.22	Wangenknochen	☐ Ja	☐ Nein
3.23	Ohren	☐ Ja	☐ Nein
3.24	Kiefergelenke	☐ Ja	☐ Nein
3.25	Oberkiefers (unterhalb der Nase)	☐ Ja	☐ Nein
3.26	Kinns	☐ Ja	☐ Nein
3.27	Unterkieferwinkels	☐ Ja	☐ Nein

3. Eiscremekopfschmerz (Kopfschmerz nach Ingestition eiskalter Nahrungsmittel) [Fortsetzung]

3.28 Bleibt der Kopfschmerz nach Verzehr eiskalter Nahrungsmittel auf der **selben** Seite und am **gleichen** Punkt des Schädels wie Ihr **bekannter, regelmäßig auftretender Kopfschmerz**? (bitte nur **EINE** Antwort ankreuzen)

☐ Ich habe **KEINEN** bekannten, regelmäßig auftretenden Kopfschmerz
☐ Seite **und** genauer Punkt sind immer **gleich**
☐ Kopfschmerz **wechselt** die Seite, ist aber am **gleichen** Punkt wie auf der Gegenseite
☐ Kopfschmerz bleibt zwar auf der **selben** Seite, tritt jedoch immer an einem **anderen** Punkt auf
☐ Kopfschmerz tritt immer an **unterschiedlichen** Punkten **und** Seiten des Schädels auf

Haben Sie eine oder mehrere der Folgenden, den Kopfschmerz <u>begleitende</u>, Symptomatiken?

3.29	Sprachstörungen	☐ Ja	☐ Nein	
3.30	Flimmersehen	☐ Ja	☐ Nein	
3.31	Sehen von Lichtblitzen	☐ Ja	☐ Nein	
3.32	Tränenfluss	☐ Ja	☐ Nein	
3.33	Rötung der Augen	☐ Ja	☐ Nein	
3.34	Nasenfluss	☐ Ja	☐ Nein	
3.35	Sensibilitätsausfälle	☐ Im Gesicht	☐ Am Körper	☐ Nein
3.36	Vorübergehende Lähmungen	☐ Im Gesicht	☐ Am Körper	☐ Nein

3.37 Genießen Sie ganzjährig eiskalte Nahrungsmittel?
☐ **ganzjährig** ☐ nur im **Sommer** ☐ nur im **Winter**

3.38 Der Kopfschmerz zeigt sich...
☐ in **kalter** <u>**und**</u> **warmer** W-itterung ☐ **nur** bei **warmer** Witterung ☐ **nur** bei **kalter** Witterung

In wie weit hat das Auftreten des Kopfschmerzes nach dem Verzehr eiskalter Nahrungsmittel ihr <u>Konsumverhalten</u> bezüglich dieser <u>beeinflusst</u>?

3.39	Ich **verzichte** auf eiskalte Nahrungsmittel	☐ Ja	☐ Nein
3.40	Ich verzehre **weniger** eiskalte Nahrungsmittel	☐ Ja	☐ Nein
3.41	Ich verzehre eiskalte Nahrungsmittel **langsam**	☐ Ja	☐ Nein
3.42	Ich schränke mein Konsumverhalten **nicht** ein und nehme gelegentliche Kopfschmerzen in Kauf	☐ Ja	☐ Nein

Leidet eines Ihrer **Familienmitglieder** an Kopfschmerzen nach dem Verzehr eiskalter Nahrungsmittel

3.43	Mutter	☐ Ja	☐ Nein	☐ Weiß nicht
3.44	Vater	☐ Ja	☐ Nein	☐ Weiß nicht
3.45	Schwester	☐ Ja	☐ Nein	☐ Weiß nicht
3.46	Bruder	☐ Ja	☐ Nein	☐ Weiß nicht
3.47	Großmutter	☐ Ja	☐ Nein	☐ Weiß nicht
3.48	Großvater	☐ Ja	☐ Nein	☐ Weiß nicht

Die Befragung ist nun abgeschlossen.
Wir bedanken uns im Namen der Klinik für Neurologie des UKK Halle für ihre Zeit und ihren Aufwand.

Wenn Sie von einer primären Kopfschmerzform betroffen sind und Interesse daran haben unsere Forschung zu unterstützen, können Sie uns unter der Emailadresse **kopfschmerzen@uk-halle.de** erreichen

2. Spezieller Teil: Kopfschmerz bei sexueller Aktivität [Fortsetzung]

2.2 **Wann tritt der Kopfschmerz assoziiert mit sexueller Aktivität auf?**
☐ Vor dem Orgasmus (Erregungsphase) ☐ Während des Orgasmus ☐ Nach dem Orgasmus

2.3 **In welchem Alter ist der Kopfschmerz assoziiert mit sexueller Aktivität das erste Mal aufgetreten?**
10er ☐ ☐ ☐ ☐ ☐ ☐ ☐ ☐ ☐ ☐
1er ☐ ☐ ☐ ☐ ☐ ☐ ☐ ☐ ☐ ☐
x0 x1 x2 x3 x4 x5 x6 x7 x8 x9

2.4 **Ist der Kopfschmerz das vorrangie Symptom?** ☐ Ja ☐ Nein

2.5 **Wie tritt der Kopfschmerz auf? (bitte nur <u>EINE</u> Antwort auswählen)**
☐ Kopfschmerzattacken (schlagartig einsetzende Kopfschmerzen)
☐ Dauerkopfschmerz (langsam steigernde und dann langanhaltende (>6h) Schmerzempfindung
☐ Episodisch wiederkehrender Kopfschmerz (Kopfschmerz beginnt und nimmt ab und dann wieder zu)

2.6 **Wie verläuft der Kopfschmerz? (bitte nur <u>EINE</u> Antwort auswählen)**
☐ Explosionsartig bei Erregungsbeginn
☐ Explosionsartig bei Orgasmus
☐ Langsam kontinuierliche Steigerung mit zunehmender Erregung

2.7 **Wie lange dauert der Kopfschmerz? (bitte nur <u>EINE</u> Antwort auswählen)**
☐ 1 Minute ☐ 30 Minuten bis 1 Stunde ☐ 1 Stunde bis 12 Stunden
☐ 12 Stunden bis 24 Stunden ☐ 24 Stunden bis 72 Stunden

2.8 Wie **intensiv** ist der Kopfschmerz **maximal** auf einer Skala von 1-10? (1= leichtester, 10= Vernichtungsschmerz)
1 ☐ ☐ ☐ ☐ ☐ ☐ ☐ ☐ ☐ ☐ 10 ☐ Weiß nicht

2.9 Wie **intensiv** ist der Kopfschmerz **durchschnittlich** über die gesamte Dauer auf einer Skala von 1-10? (1= leichtester, 10= Vernichtungsschmerz)
1 ☐ ☐ ☐ ☐ ☐ ☐ ☐ ☐ ☐ ☐ 10 ☐ Weiß nicht

2.10 **Welche Schmerzqualität hat der Kopfschmerz? (bitte nur <u>EINE</u> Antwort auswählen)**
☐ Brennend ☐ Stechend ☐ Dumpf/ Drückend
☐ Ziehend ☐ Pulsierend ☐ Hämmernd
☐ Bohrend ☐ Keine der Genannten ☐ Weiß nicht

Haben Sie eine oder mehrere der folgenden, den Kopfschmerz <u>begleitende</u>, Symptomatiken?

2.11	Sprachstörungen	☐ Ja	☐ Nein	
2.12	Flimmersehen	☐ Ja	☐ Nein	
2.13	Sehen von Lichtblitzen	☐ Ja	☐ Nein	
2.14	Tränenfluss	☐ Ja	☐ Nein	
2.15	Rötung der Augen	☐ Ja	☐ Nein	
2.16	Nasenfluss	☐ Ja	☐ Nein	
2.17	Sensibilitätsausfälle	☐ Im Gesicht	☐ Am Körper	☐ Nein
2.18	Vorübergehende Lähmungen	☐ Im Gesicht	☐ Am Körper	☐ Nein

Wo, <u>außen auf den Schädel projiziert</u>, treten die Kopfschmerzen bei sexueller Aktivität auf?
Die Kopfschmerzen treten auf im Bereich des/der...

2.19	Hinterkopfes	☐ Ja	☐ Nein
2.20	Nackens	☐ Ja	☐ Nein
2.21	Scheitels	☐ Ja	☐ Nein
2.22	Schläfen	☐ Ja	☐ Nein
2.23	Stirn	☐ Ja	☐ Nein
2.24	Oberer Nasenbereich/ Nasenwurzel	☐ Ja	☐ Nein

2. Spezieller Teil: Kopfschmerz bei sexueller Aktivität [Fortsetzung]

2.25	Augen	☐ Ja	☐ Nein
2.26	Ohren	☐ Ja	☐ Nein
2.27	Wangenknochen	☐ Ja	☐ Nein
2.28	Oberkiefers (unterhalb der Nase)	☐ Ja	☐ Nein
2.29	Kinns	☐ Ja	☐ Nein
2.30	Unterkieferwinkels	☐ Ja	☐ Nein
2.31	Kiefergelenke	☐ Ja	☐ Nein

Treten zur Zeit des Kopfschmerzes Schmerzen in anderen Teilen des Körpers auf?

2.32	Rücken	☐ Ja	☐ Nein
2.33	Nacken	☐ Ja	☐ Nein
2.34	Schulter	☐ Ja	☐ Nein
2.35	Beine	☐ Ja	☐ Nein
2.36	Arme	☐ Ja	☐ Nein

Leidet eines Ihrer Familienmitglieder an Kopfschmerz assoziiert mit sexueller Aktivität?

2.37	Vater	☐ Ja	☐ Nein	☐ Weiß nicht
2.38	Mutter	☐ Ja	☐ Nein	☐ Weiß nicht
2.39	Bruder	☐ Ja	☐ Nein	☐ Weiß nicht
2.40	Schwester	☐ Ja	☐ Nein	☐ Weiß nicht
2.41	Großvater	☐ Ja	☐ Nein	☐ Weiß nicht
2.42	Großmutter	☐ Ja	☐ Nein	☐ Weiß nicht

3. Eiscremekopfschmerz (Kopfschmerz nach Ingestition eiskalter Nahrungsmittel)

3.1 Haben Sie schon einmal Kopfschmerzen verspürt, **nachdem** Sie **eiskalte Nahrungsmittel**, in flüssiger oder fester Form, zu sich genommen haben?
☐ Nie ☐ Immer ☐ Gelegentlich

Wenn Sie hier mit "Nie" antworten, blättern Sie bitte an das Ende von Seite 6, ohne ein weiteres Kreuz zu setzen.

3.2 Seit wann ist Ihnen bekannt, dass eiskalte Nahrungsmittel bei Ihnen Kopfschmerzen auslösen können?
☐ Seit dem Erinnerungen an den Verzehr eiskalter Nahrungsmittel bestehen (ca. ab dem 6. LJ)
☐ Seit dem Übertritt in das Pubertätsalter (ca. 12. LJ)
☐ Seit dem Verlassen der Pubertät (ca. 18. LJ)

Hat eines der nachfolgenden Nahrungsmittel bei Ihnen schon einmal Kopfschmerzen ausgelöst?

3.3	Eiscreme	☐ Ja	☐ Nein	☐ Nie verzehrt
3.4	Frozen Yoghurt	☐ Ja	☐ Nein	☐ Nie verzehrt
3.5	Eiskalte Getränke	☐ Ja	☐ Nein	☐ Nie verzehrt
3.6	Getränke mit Crushed Ice z.B. Cocktails	☐ Ja	☐ Nein	☐ Nie verzehrt
3.7	Milchshakes/ Eiskaffee/ -Schokolade	☐ Ja	☐ Nein	☐ Nie verzehrt
3.8	Getränke aus flüssigem Eisschnee (Slush-Ice, geeiste Fruchtsmoothies, Frozen Daiquiris)	☐ Ja	☐ Nein	☐ Nie verzehrt
