# Supplementary material for: In the heat of the moment: prevalence and clinical characteristics of headache associated with sexual activity (head HEATS study)
Source: J Headache Pain. 2026 Sep 29;27(1):228. doi: 10.1186/s10194-026-02516-7 (PMC13625492; doi:10.1186/s10194-026-02516-7)
Supplement: Supplementary file 2 [file 10194_2026_2516_MOESM2_ESM.pdf]

Study: **Determination of the frequency, characteristics, and potential pathophysiological relationships of rare primary headache types**

Department of Neurology, University Hospital Halle (Saale)

Please use a ballpoint pen (black or blue) to fill out this questionnaire. This questionnaire is evaluated automatically by an optical scanner. Please ensure crosses are marked clearly within the designated boxes for optimal data recording.

Dear Participant,

With the following anonymous questionnaire, we would like to survey you within the framework of a doctoral thesis regarding the frequency of primary headaches. The main focus will be on headache associated with sexual activity (HAS) and ice cream headache (cold-stimulus headache). We hope that your information will help us better understand the frequency and causes of these headache forms.

Participation in this survey is voluntary. This is an anonymous questionnaire, and its data will be collected and evaluated at the Department of Neurology, University Hospital Halle. When publishing the study results, it will be impossible to trace any data back to your personal identity. By filling out and submitting or returning the questionnaire, you consent to having your data collected and scientifically evaluated.

Please fill out the questionnaire only once to avoid skewing the study results. If you have any questions regarding this study, you can contact us at any time. Thank you for your cooperation!

(The responsible Ethics Committee of the Medical Faculty of the Martin Luther University Halle-Wittenberg has raised no objections to the conduction of this study.)

Sincerely,

Dr. med. Torsten Kraya (Senior Physician, Head of the Headache Outpatient Clinic)

Department of Neurology, University Hospital Halle (Saale), Ernst-Grube-Str. 40, 06097 Halle (Saale), Germany

Phone: +49 (0)345 557-3340 | Fax: +49 (0)345 557-3335

1. General Information

1.1 Please encode your current age as explained in the introduction: [ _____ ]

1.2 What is your biological sex?

[ ] Female

[ ] Male

1.3 Do you regularly experience similar types of headaches?

[ ] Never

[ ] Very rarely (once every 3 months)

[ ] Rarely (1–6 times per month)

[ ] Frequently (7–15 times per month)

[ ] Daily

Do you knowingly suffer from any of the following headache forms?

1.4 Migraine: [ ] Yes | [ ] No

1.5 Cluster headache: [ ] Yes | [ ] No

1.6 Cough headache: [ ] Yes | [ ] No

1.7 Tension-type headache: [ ] Yes | [ ] No

1.8 Trigeminal neuralgia: [ ] Yes | [ ] No

1.9 Primary exercise headache: [ ] Yes | [ ] No

1.10 SUNCT headache: [ ] Yes | [ ] No

1.11 Thunderclap headache: [ ] Yes | [ ] No

1.12 Paroxysmal hemicrania: [ ] Yes | [ ] No

1.13 Since when have you been suffering from one of the above-mentioned headaches?

[ ] I do not suffer from any of the above

[ ] Since memory began (approx. from age 6)

[ ] Since entering puberty (approx. age 12)

[ ] Since leaving puberty (approx. age 18)

Does any member of your family suffer from a primary headache disorder?

1.14 Mother: [ ] Yes | [ ] No | [ ] Don't know

1.15 Father: [ ] Yes | [ ] No | [ ] Don't know

1.16 Sister: [ ] Yes | [ ] No | [ ] Don't know

1.17 Brother: [ ] Yes | [ ] No | [ ] Don't know

1.18 Grandmother: [ ] Yes | [ ] No | [ ] Don't know

1.19 Grandfather: [ ] Yes | [ ] No | [ ] Don't know

Do you knowingly suffer from any of the following medical conditions?

1.20 Asthma: [ ] Yes | [ ] No

1.21 Coronary artery disease: [ ] Yes | [ ] No

1.22 Depression: [ ] Yes | [ ] No

1.23 Malignancies (cancer): [ ] Yes | [ ] No

1.24 Arterial hypertension (high blood pressure): [ ] Yes | [ ] No

1.25 Anxiety disorder: [ ] Yes | [ ] No

Do you take any of the following medications for the prevention of migraine attacks?

1.26 Beta-blockers (Metoprolol / Propranolol): [ ] Yes | [ ] No

1.27 Flunarizine (Flunavert / Natil N): [ ] Yes | [ ] No

1.28 Topiramate (Topimac / Topimax / Topiragamma): [ ] Yes | [ ] No

1.29 Amitriptyline (Amineurin / Saroten / Syneudon): [ ] Yes | [ ] No

1.30 Valproic acid (Orfiril): [ ] Yes | [ ] No

Do you take any of the following pain medications during the acute phase of a headache?

1.31 Aspirin / ASA: [ ] Yes | [ ] No

1.32 Ibuprofen (Dolormin / Ibudolor / Dolgit): [ ] Yes | [ ] No

1.33 Naproxen: [ ] Yes | [ ] No

1.34 Diclofenac (Diclac / Voltaren): [ ] Yes | [ ] No

1.35 Phenazone (Migräne-Kranit): [ ] Yes | [ ] No

1.36 Metamizole (Novalgin / Novaminsulfon): [ ] Yes | [ ] No

1.37 Paracetamol: [ ] Yes | [ ] No

1.38 ASA + Paracetamol + Caffeine (Thomapyrin / Dolopyrin / Neuralgin / Ratiopyrin): [ ] Yes | [ ] No

1.39 Triptans (Sumatriptan / Eletriptan / etc.): [ ] Yes | [ ] No

2. Special Section: Headache Associated with Sexual Activity (HAS)

2.1 Do you experience headaches before, during, or after sexual intercourse?

[ ] Never

[ ] Occasionally

[ ] Always

(If you answer "Never" here, please skip to the "Ice Cream Headache" section on page 4).

2.2 When does the headache associated with sexual activity occur?

[ ] Before orgasm (arousal phase)

[ ] During orgasm

[ ] After orgasm

2.3 At what age did the headache associated with sexual activity occur for the first time? [ _______ ] years

2.4 Is the headache the predominant symptom? [ ] Yes | [ ] No

2.5 How does the headache present? (Please select ONLY ONE answer)

[ ] Single headache attacks

[ ] Continuous headache (slowly increasing and then long-lasting >6h pain sensation)

[ ] Episodically recurring headache (headache starts, subsides, and recurs)

2.6 How does the headache progress? (Please select ONLY ONE answer)

[ ] Explosive onset at the beginning of arousal

[ ] Explosive onset at orgasm

[ ] Slow, continuous increase with increasing sexual arousal

2.7 How long does the headache last? (Please select ONLY ONE answer)

[ ] < 1 minute

[ ] 1 minute to 30 minutes

[ ] 30 minutes to 1 hour

[ ] 1 hour to 12 hours

[ ] 12 hours to 24 hours

[ ] 24 hours to 72 hours

2.8 How intense is the headache maximally on a scale of 1–10? (1 = mildest, 10 = excruciating pain / thunderclap pain) [ ___ ] | [ ] Don't know

2.9 How intense is the headache on average over the entire duration on a scale of 1–10? (1 = mildest, 10 = excruciating pain) [ ___ ] | [ ] Don't know

2.10 What is the quality of the headache? (Please select ONLY ONE answer)

[ ] Burning

[ ] Stabbing

[ ] Dull / Pressing

[ ] Pulling / Drawing

[ ] Boring

[ ] Pulsating

[ ] Hammering

[ ] None of the above

[ ] Don't know

Do you experience one or more of the following symptoms accompanying the headache?

2.11 Speech disturbances: [ ] Yes | [ ] No

2.12 Flickering vision / Scintillating scotoma: [ ] Yes | [ ] No

2.13 Seeing flashes of light: [ ] Yes | [ ] No

2.14 Tearing (Lacrimation): [ ] Yes | [ ] No

2.15 Redness of the eyes (Conjunctival injection): [ ] Yes | [ ] No

2.16 Nasal discharge (Rhinorrhea): [ ] Yes | [ ] No

2.17 Sensory disturbances: [ ] In the face | [ ] On the body | [ ] No

2.18 Temporary paralysis: [ ] In the face | [ ] On the body | [ ] No

Where, projected externally onto the skull, do the headaches associated with sexual activity occur? (The headaches occur in the area of...)

2.19 Back of the head (Occipital): [ ] Yes | [ ] No

2.20 Neck (Nuchal): [ ] Yes | [ ] No

2.21 Crown (Parietal): [ ] Yes | [ ] No

2.22 Temples (Temporal): [ ] Yes | [ ] No

2.23 Forehead (Frontal): [ ] Yes | [ ] No

2.24 Upper nasal area / Root of the nose: [ ] Yes | [ ] No

2.25 Eyes (Orbital): [ ] Yes | [ ] No

2.26 Ears: [ ] Yes | [ ] No

2.27 Cheekbones: [ ] Yes | [ ] No

2.28 Upper jaw (below the nose): [ ] Yes | [ ] No

2.29 Chin: [ ] Yes | [ ] No

2.30 Angle of the jaw: [ ] Yes | [ ] No

2.31 Temporomandibular joints: [ ] Yes | [ ] No

The survey is now concluded. We thank you on behalf of the Department of Neurology at the University Hospital Halle for your time and effort! If you are affected by a primary headache and wish to support our research, you can reach us at kopfschmerzen@uk-halle.de.
