# Supplementary material for: In the heat of the moment: prevalence and clinical characteristics of headache associated with sexual activity (head HEATS study)
Source: J Headache Pain. 2026 Sep 29;27(1):228. doi: 10.1186/s10194-026-02516-7 (PMC13625492; doi:10.1186/s10194-026-02516-7)
Supplement: Supplementary file 3 [file 10194_2026_2516_MOESM3_ESM.pdf]

Submission ID: ac22a56f-a151-418d-9839-84af48b2ae57

Manuscript Title: **In the Heat of the Moment: Prevalence and Clinical Characteristics of Headache Associated with Sexual Activity (Head HEATS study)**

Journal: The Journal of Headache and Pain

Corresponding Author: Dr. med. Simon Heintz

STROBE Statement—Checklist of items that should be included in reports of cross-sectional studies

**Item No**	**Recommendation**	**Where to find in Manuscript (Section / Page)**
Title and abstract		
1	(a) Indicate the study's design with a commonly used term in the title or the abstract	Title / Abstract (Background & Methods)
	(b) Provide a informative and balanced summary of what was done and found in the abstract	Abstract (Background, Objectives, Methods, Results, Conclusions)
Introduction		
2	Background / rationale: Explain the scientific background and rationale for the investigation being reported	Introduction (Paragraph 1–3)
3	Objectives: State specific objectives, including any prespecified hypotheses	Introduction (Paragraph 4 / final objective statement)
Methods		

Item No	Recommendation	Where to find in Manuscript (Section / Page)
4	Study design: Present key elements of study design early in the paper	Methods (Study Design)
5	Setting: Describe the setting, locations, and relevant dates, including periods of recruitment, exposure, follow-up, and data collection	Methods (Study Design & Cohort)
6	Participants: (a) Give the eligibility criteria, and the sources and methods of selection of participants	Methods (Cohort)
7	Variables: Clearly define all outcomes, exposures, predictors, potential confounders, and effect modifiers. Give diagnostic criteria, if applicable	Methods (Questionnaire & Data collection)
8	Data sources / measurement: For each variable of interest, give sources of data and details of methods of assessment (measurement). Describe comparability of assessment methods if there is more than one group	Methods (Questionnaire & EvaSys platform)
9	Bias: Describe any efforts to address potential sources of bias	Methods (Data collection and analysis / Limitations in Discussion)
10	Study size: Explain how the study size was arrived at	Methods (Cohort & Flowchart description)

Item No	Recommendation	Where to find in Manuscript (Section / Page)
11	Quantitative variables: Explain how quantitative variables were handled in the analyses. Describe which groupings were chosen and why	Methods (Data collection and analysis)
12	Statistical methods: (a) Describe all statistical methods, including those used to control for confounding	Methods (Data collection and analysis - mentioning Mann-Whitney U test, Chi-square, Fisher's exact test,)
	(b) Describe any methods used to examine subgroups and interactions	Methods (Data collection and analysis)
	(c) Explain how missing data were addressed	Methods (Data collection and analysis: exclusion of incomplete demographic records)
	(d) If applicable, describe analytical methods taking account of sampling strategy	Methods (Data collection and analysis)
	(e) Describe any sensitivity analyses	Methods (Data collection and analysis: sensitivity analysis)
Results		
13	Participants: (a) Report numbers of individuals at each stage of study—e.g., numbers potentially eligible, examined for eligibility, confirmed	Results (Sample characteristics & Flowchart Fig. 1)

Item No	Recommendation	Where to find in Manuscript (Section / Page)
	eligible, included in the study, completing follow-up, and analysed	
	(b) Give reasons for non-participation at each stage	Results (Sample characteristics / Flowchart)
	(c) Consider use of a flow diagram	Results / Figure 1 (Flow chart)
14	Descriptive data: (a) Give characteristics of study participants (e.g., demographic, clinical, social) and information on exposures and potential confounders	Results (Sample characteristics & Table 1)
	(b) Indicate number of participants with missing data for each variable of interest	Results (Sample characteristics text)
15	Outcome data: Report numbers of outcome events or summary measures	Results (Prevalence of HAS, Table 1, Table 3)
16	Main results: (a) Give unadjusted estimates and, if applicable, confounder-adjusted estimates and their precision (e.g., 95% confidence intervals). Make clear which confounders were adjusted for and why	Results (Co-occurrences, Table 3)
	(b) Report category boundaries when continuous variables were categorized	Results (Age distribution & Table 3)

Item No	Recommendation	Where to find in Manuscript (Section / Page)
	(c) If relevant, translate estimates of relative risk into absolute risk for a meaningful time period	N/A (Cross-sectional prevalence study)
17	Other analyses: Report other analyses done—e.g., analyses of subgroups and interactions, and sensitivity analyses	Results (Pain intensity and co-occurrence comparison)
Discussion		
18	Key results: Summarize key results with reference to study objectives	Discussion (Paragraph 1)
19	Limitations: Discuss limitations of the study, taking into account sources of potential bias or imprecision. Discuss both direction and magnitude of any potential bias	Discussion (Strengths and limitations section)
20	Interpretation: Give a cautious overall interpretation of results considering objectives, limitations, multiplicity of analyses, and results from similar studies	Discussion (Prevalence, Phenotype, Co-occurrence sections & Conclusions)
21	Generalisability: Discuss the generalisability (external validity) of the study results	Discussion (Strengths and limitations section)
Other information		

Item No	Recommendation	Where to find in Manuscript (Section / Page)
22	Funding: Give source of funding and the role of the funders for the present study	Statements and Declarations (Page 21 / end of manuscript)
